# Chronic viral infections impinge on naive bystander CD8 T cells

**DOI:** 10.1002/iid3.300

**Published:** 2020-03-26

**Authors:** Isabel Barnstorf, Suzanne P. M. Welten, Mariana Borsa, Nicolas S. Baumann, Katharina Pallmer, Nicole Joller, Roman Spörri, Annette Oxenius

**Affiliations:** ^1^ Institute of Microbiology, Department of Biology ETH Zürich Zürich Switzerland; ^2^ Institute of Experimental Immunology University of Zürich Zürich Switzerland

**Keywords:** CD8 T cells, chronic virus infection, LCMV, naive bystander T cells, T‐cell primin

## Abstract

**Introduction:**

Epidemiological data suggest that persistent viral infections impair immune homeostasis and immune responsiveness. Previous studies showed that chronic virus infections negatively impact bystander T‐cell differentiation and memory formation but there is limited knowledge of how chronic virus infections impinge on heterologous naive T‐cell populations.

**Methods:**

We used adoptive transfer of naive CD8 T cells with defined nonviral specificity into hosts, which were subsequently chronically infected with lymphocytic choriomeningitis virus, followed by analyses of numeric, phenotypic, and functional changes provoked in the chronically infected host.

**Results:**

We demonstrate that chronic virus infections have a profound effect on the number and phenotype of naive bystander CD8 T cells. Moreover, primary expansion upon antigen encounter was severely compromised in chronically infected hosts. However, when naive bystander CD8 T cells were transferred from the chronically infected mice into naive hosts, they regained their expansion potential. Conversely, when chronically infected hosts were supplied with additional antigen‐presenting cells (APCs), primary expansion of the naive CD8 T cells was restored to levels of the uninfected hosts.

**Conclusions:**

Our results document numeric, phenotypic, and functional adaptation of bystander naive CD8 T cells during nonrelated chronic viral infection. Their functional impairment was only evident in the chronically infected host, indicating that T‐cell extrinsic factors, in particular the quality of priming APCs, are responsible for the impaired function of naive bystander T cells in the chronically infected hosts.

## Graphical abstract

We investigated how chronic virus infections impinge on heterologous naive T‐cell populations, using adoptive transfers of naive CD8 T cells with defined nonviral specificity. Naive bystander CD8 T cells were numerically reduced, phenotypically altered, and their primary expansion upon antigenic rechallenge was severely compromised in chronically infected hosts. However, the impaired expansion potential was only evident in the chronically infected host, indicating that T‐cell extrinsic factors are responsible for this impairment.

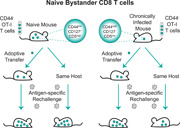

## 1 INTRODUCTION

During primary T‐cell responses, activated T cells clonally expand and form an effector pool as well as a long‐lived memory population.^1^, ^2^ There is epidemiological evidence that persistent viral infections like hepatitis C virus (HCV), human immunodeficiency virus (HIV), and lymphocytic choriomeningitis virus (LCMV) infection in mice functionally impair CD8 T cells in their immune responsiveness.^3^ Chronic infections with actively replicating viruses, such as HCV, HIV, and LCMV, result in virus‐specific CD8 T‐cell responses that are generally compromised in size and function (termed T‐cell exhaustion).^4^ However, heterologous immunity (the CD8 T‐cell response unrelated to the virus or the "bystander" cells) has also been reported to be affected by chronic virus infections. For instance, LCMV infection induces a sustained loss of bystander memory T cells,^5^, ^6^ and negatively impacts memory development of the antigen‐unspecific CD8 T cells.^7^ Bystander memory T cells in HIV‐infected individuals also acquire an activated phenotype.^8^ The naive T‐cell compartment can be altered by chronic viral infections as well. In patients with chronic HCV infection, naive T cells express phenotypical markers that are associated with memory and diminished levels of CD5,^9^ which correlates with a lower threshold for T‐cell receptor (TCR) signaling. This effect was reported to be transient, since these alterations could be reversed within 2 years following successful therapy.^9^ Chronic virus infections also impair T‐cell differentiation of tumor‐specific CD8 T cells, leading to a diminished tumor control.^10^

We examined the effects of chronic virus infections on a defined population of naive bystander CD8 T cells. We show that viral infections can have a profound effect on the numbers, phenotype, and expansion potential of naive bystander CD8 T cells. However, these impairments can be reversed when bystander cells are transferred into uninfected hosts, indicating that T‐cell extrinsic cues caused by the chronic virus restrict their functional potential.

## 2 MATERIALS AND METHODS

### 2.1 Mice

C57BL/6J (CD45.2^+^) mice were purchased from Janvier Elevage. Maxi mice express a TCR specific for MCMV‐specific M38_316‐323_/K^b^ ^11^ and OT‐I mice express a TCR specific for OVA_257‐264_/K^b^.^12^ Both Maxi and OT‐I mice are on a CD45.1 background. All mice were housed and bred under specific pathogen‐free conditions at the ETH Phenomics Center. Mice were used between 7 and 12 weeks of age and were age‐ and sex‐matched. Animal experiments were performed according to the institutional policies and Swiss federal regulations, and were approved by the cantonal veterinary office of Zürich (animal experimentation permissions: 228/2013, 166/2016).

### 2.2 Viruses and infections

LCMV WE and Docile were provided by Dr. R. M. Zinkernagel and were propagated at a low multiplicity of infection on L929 fibroblast cells (WE) or Madin‐Darby Canine Kidney endothelial cells (Docile). For chronic infection, mice were infected intravenously (IV) with 2 × 10^6^ ffu LCMV Docile and for an acute infection, mice were infected IV with 2 × 10^2^ ffu LCMV WE. Recombinant MCMV lacking m157 (MCMVΔm157) is described^13^ and is referred to as MCMV. MCMV was propagated on M2‐10B4 cells as previously described.^14^ Mice were infected IV with 10^6^ plaque‐forming units (PFU). Recombinant Vaccinia virus (Western Reserve) encoding ovalbumin (VV‐OVA) inserted into the thymidine kinase gene was grown on BSC40 cells and was provided by Dr. P. Klenerman. VV‐OVA was grown on BSC40 cells at a low multiplicity of infection. Mice were infected intraperitoneally with 10^6^ PFU.

### 2.3 Flow cytometry and lymphocyte stimulation

Single‐cell suspensions were prepared from the spleen by mincing the tissue through a 70 µm cell strainer using a syringe plunger. For red blood cell lysis, samples were treated with ammonium‐chloride‐potassium lysis buffer for 1 minute at room temperature. To detect naive bystander CD8 T cells in the spleen, CD8 T cells were enriched using anti‐CD8α MACS beads (Miltenyi Biotech) according to the manufacturer's protocol. Cells from the lungs were isolated as described.^6^ Cell surface staining was performed for 20 minutes at 4°C in phosphate buffer saline (PBS) containing 2% fetal calf serum (FCS) and 5 mM ethylenediaminetetraacetic acid. Intracellular cytokines were detected as described.^6^ Fluorophore‐conjugated antibodies were purchased from BioLegend (Lucerna Chem) or eBioscience. The following antibodies (clone) were used: CD8α (53‐6.7), CD127 (A7R34), CD45.1 (A20), CD62L (MEL‐14), CD44 (IM7), CD5 (53‐7.3), KLRG1 (2F1), interferon γ (IFNγ) (XMG1.2), tumor necrosis factor (TNF) (MP6‐XT22), interleukin‐2 (IL‐2) (JES6‐5H4). Live/Dead Fixable near‐IR (Life Technologies) was used to exclude dead cells. OVA_257‐264_ (K^b^) monomer was produced as described^15^ and was tetramerized using Streptavidin‐APC. Flow cytometric acquisition was performed using LSRII or Fortessa flow cytometer (BD Biosciences) and FACSDiva software. Data were analyzed using FlowJo software (Tree Star). Representative gating strategy is shown in Figure S2.

### 2.4 T‐cell isolation and adoptive transfer

CD8 T cells from the spleen of naive OT‐I or Maxi mice were purified using anti‐CD8α MACS beads (Miltenyi Biotech) according to the manufacturer's instructions. Cells were stained as described above and sorted for CD8 CD45.1^+^ CD62L^+^ CD44^–^ using a BD FACSAria Sorter. A total of 10^6^ naive OT‐I or Maxi T cells were transferred into CD45.2^+^ hosts. To isolate naive bystander cells, CD8 T cells were purified from the spleen using anti‐CD8α MACS beads. Cells were stained as described and sorted for CD8α and CD45.1. To determine the fold expansion, the number of OT‐I T cells after VV‐OVA challenge (or peptide administration) was determined in each mouse in the spleen and divided by the mean number of OT‐I T cells that was determined in the spleen of a separate cohort of LCMV‐infected and naive mice on the day of the VV‐OVA (or peptide) challenge (day 30 after LCMV infection).

### 2.5 Peptide vaccinations and BMDC transfer

Mice were vaccinated IV with 50 µg OVA_257‐264_‐peptide (EMC mirocollections) combined with 20 µg CpG‐1668 (Mircosynth) in PBS. For priming of endogenous OVA_257‐264_‐specific CD8 T cells, mice were vaccinated subcutaneously with 50 µg OVA_257‐264_‐peptide in alum. Bone marrow‐derived dendritic cells (BMDCs) were generated by culturing bone marrow cells for 8 to 10 days in Roswell Park Memorial Institute (Life Technologies) supplemented with 10% FCS (Omnilab), 2 mM l‐glutamine (Gibco) and 1% penicillin/streptomycin (Gibco) in the presence of 200 ng/mL Flt3L (Life Technologies) at 37°C. BMDCs were matured overnight with 100 ng/mL lipopolysaccharide. The following day BMDCs were loaded with OVA_257‐264_‐peptide for 2 hours and were washed to remove free peptide. Between 0.75 and 1.25 × 10^6^ loaded BMDCs were IV injected into mice.

### 2.6 Statistical analysis

Statistical significance was determined using GraphPad Prism with *P < .05. Statistical tests are indicated for each figure.

## 3 RESULTS AND DISCUSSION

### 3.1 Chronic virus infection reduces the number and alters the phenotype of naive bystander T cells

**Figure 1:**
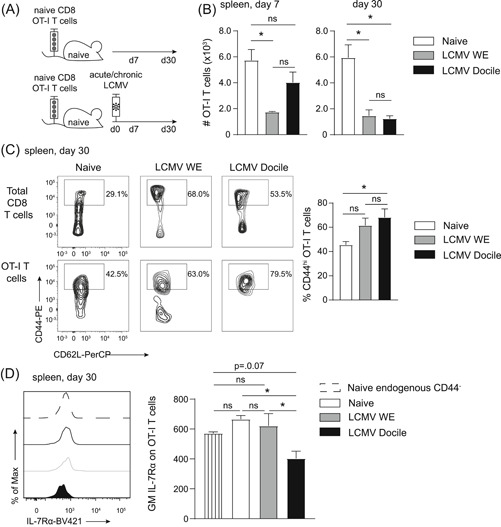
Lymphocytic choriomeningitis virus (LCMV) infection reduces the abundance and alters the phenotype of naive bystander T cells. A, Experimental setup: 10^6^ CD44^−^ CD62L^+^ CD45.1^+^ OT‐I T cells were adoptively transferred into wild‐type (CD45.2^+^) mice. One day after transfer, recipient mice were infected with 200 ffu LCMV WE (acute infection) or 2 × 10^6^ ffu LCMV Docile (chronic infection). The total number and phenotype of OT‐I T cells were determined 7 and 30 days after infection. B, Total number of OT‐I T cells in the spleen at 7 and 30 days after infection. Data are pooled from two independent experiments with three to five mice per group. C, Expression of CD44 on total CD8 T cells (excluding OT‐I T cells) and on OT‐I T cells in the spleen are shown. Bar graphs represent the percentage of CD44^hi^ OT‐I T cells. D, Histogram shows expression of interleukin‐7Rα (IL‐7Rα) on OT‐I T cells in the spleen. The bar graph shows the geometric mean of IL‐7Rα on OT‐I T cells in the spleen. For comparison, CD44^−^ CD8 T cells from naive mice are included. C and D, One out of two representative experiments is shown with three to five mice per group. Statistical analyses were performed using the Kruskal‐Wallis test with Dunn's posthoc test (B and C) or one‐way analysis of variance with Tukey's posthoc test (D), *P < .05 and not significant (NS); *P* ≥ .05. Bar graphs represent mean + SEM

To determine the effect of chronic virus infections on naive bystander CD8 T cells, naive (CD44^low^/CD62L^+^) CD45.1^+^ TCR transgenic OVA_257‐264_/K^b^‐specific OT‐I CD8 T cells were adoptively transferred into wild‐type CD45.2^+^ mice, followed by acute or chronic infection with LCMV (Figure 1A). In this setting, the TCR of OT‐I T cells is not engaged, as LCMV does not encode the OVA_257‐264_ epitope and the OT‐I TCR is not cross‐reactive towards LCMV‐encoded antigens.^6^ Seven days after infection, the number of naive bystander OT‐I T cells in the spleen was severely reduced upon acute LCMV infection. The number of OT‐I T cells had declined in chronically infected mice 30 days after infection, but no further decline was observed in acutely infected hosts at this time (Figure 1B). We performed similar experiments where mice were infected with murine cytomegalovirus (MCMV), which causes a latent infection with sporadic stages of reactivation (Figure S1A). However, no reduction in the naive bystander T‐cell pool was observed (Figure S1B), indicating that this numeric reduction is LCMV‐specific. In both MCMV and LCMV infection, remodeling of the splenic architecture occurs although with different kinetics^16^, ^17^ and via distinct mechanisms. In LCMV infection, infected fibroblastic reticular cells are killed by CD8 T cells,^17^ which could potentially compromise survival niches for naive CD8 T cells leading to their numerical reduction.

With respect to the phenotype of the bystander OT‐I T cells, we observed an increased expression of CD44 and a diminished expression of the IL‐7Rα (CD127) upon chronic LCMV infection (Figure 1C,D), while only slight changes were observed in acute LCMV infection. This alteration in expression of CD44 was also observed when naive bystanders were exposed to MCMV infection (Figure S1C). Moreover, we could confirm our findings in chronic LCMV infection using TCR transgenic CD8 T cells specific for the MCMV M38_316‐323_/K^b^ antigen (Maxi) as naive bystanders (Figure S1D‐G).^11^ In HCV‐infected individuals, naive bystander T cells express lower levels of CD5 compared with healthy individuals.^9^ In line with this, we observed reduced CD5 expression on naive bystander Maxi cells in the spleen of hosts chronically infected with LCMV compared with controls (Figure S1H). Combined, these data indicate that chronic LCMV infection reduces the number of naive bystander T cells and alters their phenotype.

### 3.2 Chronic virus infection compromises naive bystander T cells in expansion

**Figure 2:**
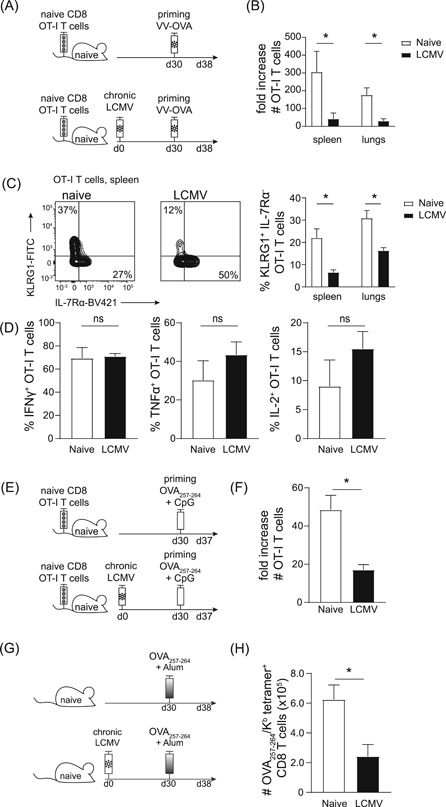
Chronic virus infections compromise naive bystander T cells in their ability to expand. A, Experimental setup: 10^6^ naive OT‐I T cells were adoptively transferred into hosts that were chronically infected with LCMV the following day. Thirty days after infection mice were infected with 10^6^ plaque‐forming units VV‐OVA. Analysis of OT‐I T cells 8 days after priming. LCMV uninfected hosts served as control. B, Fold increase of naive OT‐I T cells at day 8 after VV‐OVA infection. C, Flow cytometry plots show expression of KLRG1/interleukin‐7Rα (IL‐7Rα) on OT‐I T cells in the spleen. Bar graphs show the percentage of OT‐I T cells that are KLRG1^+^/IL‐7Rα^−^ in the spleen and the lungs. D, interferon γ (IFNγ), tumor necrosis factor α (TNF‐α), and IL‐2 production by OT‐I T cells in the spleen is shown. B‐D, Pooled data of three (B and C) or two (D) experiments is shown with two to four mice/group/experiment. E, Experimental setup: 10^6^ naive OT‐I CD8 T cells were adoptively transferred into hosts that were chronically infected with LCMV the following day. Thirty days after infection mice were vaccinated with OVA_257‐264_‐peptide + CpG, and the response was analyzed 7 days after priming. F, Fold increase of OT‐I T cells on day 7 after priming. The pooled data of four experiments is shown with three to five mice per group. G, Experimental setup: mice were chronically infected with LCMV. Thirty days after infection mice were vaccinated with OVA_257‐264_‐peptide + alum and 8 days later the OVA_257‐264_/K^b^‐specific CD8 T cells response was determined in the spleen by tetramer staining. H, Total number of OVA_257‐264_/K^b^‐specific CD8 T cells in the spleen. F‐H, One experiment out of two is shown with five mice per group. Statistical analyses were determined using the Mann‐Whitney test, *P < .05 and not significant (NS); *P* ≥ .05. Bar graphs represent mean + SEM

Both acute and chronic LCMV infection induced a numeric reduction in the pool of naive bystander cells. However, phenotypical alterations were mostly observed in chronic infection, therefore we focused on this model. Next, we addressed whether the ability of LCMV‐exposed naive bystander CD8 T cells to respond to the cognate antigen is impaired, by infecting the hosts with VV‐OVA and thereby priming the bystander OT‐I T cells (Figure 2A). The OT‐I T cells that had been exposed to chronic LCMV infection expanded poorly in the spleen and lung (Figure 2B). In addition, the fraction of KLRG1^+^ OT‐I T cells, which marks short‐lived effector cells, was decreased in LCMV‐infected compared with control hosts (Figure 2C), indicating that effector cell formation was impaired. Nevertheless, no differences were found in the production of IFNγ, TNF, and IL‐2 (Figure 2D) by OT‐I T cells upon restimulation with the cognate antigen. These data indicate that chronic LCMV infection severely compromises naive bystander CD8 T cells in their ability to expand upon priming and to a minor extent in differentiating into effector cells.

Chronic infection can impair the replication of VV,^6^ which might explain the observed effect. To circumvent this caveat, we immunized mice with OVA_257‐264_‐peptide in combination with CpG (Figure 2E). Also in this setting, naive bystander OT‐I T cells expanded to a much smaller extent when exposed to a chronic virus infection (Figure 2F). Similarly, when LCMV‐exposed mice were immunized with OVA_257‐264_‐peptide, reduced numbers of endogenous OVA_257‐264_‐specific CD8 T cells were detected compared with controls (Figure 2G,H). Combined, these results show that monoclonal and polyclonal naive CD8 T cells are hampered in their ability to expand when priming occurs in chronically infected hosts.

### 3.3 LCMV‐exposed naive bystander T‐cell impairment is due to T‐cell extrinsic factors

**Figure 3:**
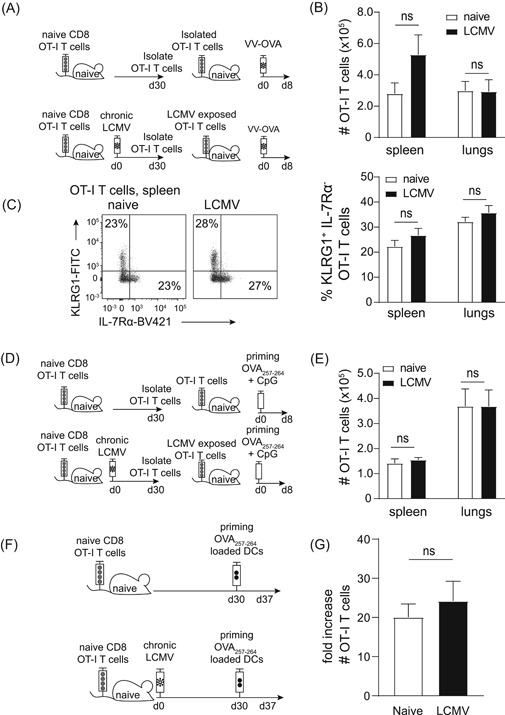
Impaired T‐cell expansion in chronic virus infection is T‐cell extrinsic. A, Experimental setup: 10^6^ naive OT‐I T cells were transferred into hosts that were chronically infected with LCMV the following day. Thirty days after infection, OT‐I T cells were sorted from infected or uninfected hosts and 10^3^ cells were transferred into new hosts that were infected 1 day later with VV‐OVA. B, Numbers of OT‐I T cells at day 8 after priming are shown for the spleen and the lungs. C, Fluorescence‐activated cell sorting plots show expression of KLRG/interleukin‐7Rα (IL‐7Rα) on OT‐I T cells in the spleen. The bar graph shows the percentage of OT‐I T cells that is KLRG1^+^/IL‐7Rα^−^ in the spleen and the lungs. D, Experimental setup: 10^6^ naive OT‐I T cells were transferred into hosts that were chronically infected with LCMV the following day. Thirty days after infection, OT‐I cells were sorted from infected or uninfected hosts and 10^3^ cells were transferred into new hosts, followed by immunization with OVA_257‐264_‐peptide + CpG. E, Numbers of OT‐I cells 8 days after priming are shown. F, Experimental setup: naive OT‐I T cells were adoptively transferred into hosts that were chronically infected with LCMV the following day. Thirty days after infection, mice received bone marrow‐derived dendritic cells loaded with OVA_257‐264_‐peptide and 7 days later the fold expansion of OT‐I T cells was determined in the spleen. G, Bar graphs represent mean + SEM. B‐F, One experiment out of four (B‐C) or three (E) is shown with three to five mice/group/experiment. G, Pooled data from three experiments is shown with three to five mice/group/experiment. Statistical analyses were determined using the Mann‐Whitney test, NS; *P* ≥ .05

Next, we determined whether the compromised naive bystander T‐cell expansion was due to T‐cell intrinsic or extrinsic factors. We isolated LCMV‐exposed or nonexposed bystander OT‐I T cells 30 days after infection and transferred equal numbers into naive hosts that were subsequently infected with VV‐OVA (Figure 3A). The OT‐I T cells that had been exposed to chronic LCMV infection expanded to comparable levels as OT‐I T cells isolated from naive hosts (Figure 3B). In addition, a normal ability for effector cell differentiation, as measured by KLRG1 expression, was observed for LCMV‐exposed OT‐I T cells (Figure 3C), despite having an altered phenotype before transfer (Figure 1C,D). Together, these data indicate that cell‐extrinsic factors, induced by a chronic virus infection, restrain naive T cells in their expansion and effector cell differentiation during priming. To corroborate this finding, we immunized secondary hosts that received LCMV‐exposed OT‐I T cells with OVA_257‐264_‐peptide + CpG (Figure 3D). These OT‐I T cells expanded to the same level as their unexposed counterparts (Figure 3E), corroborating the previous results.

Dendritic cells (DCs) are central for the priming of naive T cells and these cells are functionally altered in chronic LCMV infection.^10^, ^18^ We addressed if we could rescue T‐cell priming in chronically infected hosts by providing OVA_257‐264_‐peptide‐loaded DCs derived from the bone marrow of naive mice. Strikingly, OT‐I T cells expanded to a similar extent in naive and chronically infected hosts when exogenous DCs were provided (Figure 3F,G). This suggests that naive T cells are hampered in their ability to expand when priming occurs in chronically infected hosts, but this deficiency can be overcome when a proper priming signal is provided.

## 4 CONCLUSION

Our findings highlight the consequences of chronic viral infections on de novo induction of heterologous immunity. The reduced responsiveness of bystander naive CD8 T cells towards their cognate antigen is largely due to T‐cell extrinsic factors associated with chronic LCMV infection, rather than imprinting of T‐cell intrinsic functional alterations. These findings are in line with a recent report that showed that antitumor T‐cell responses are skewed in chronically infected hosts, but this can be overcome when strong stimulatory antigen‐presenting cells (APCs) are given.^10^ There are several reports that show that chronic virus infections modulate APC function. For instance, LCMV can infect DCs leading to impaired maturation, hampered antigen presentation capacity,^19^ and inability to stimulate T‐cell proliferation.^20^ Furthermore, high levels of type I IFNs which are produced during chronic LCMV infection, target DCs to induce the immunosuppressive factors IL‐10 and PD‐L1.^18^

Tumor‐specific T‐cell responses that develop in the presence of a chronic LCMV infection exhibit diminished T‐bet and granzyme B expression, indicating that these cells are altered in effector cell formation.^10^ We also observed a diminished effector cell formation, indicated by diminished KLRG1 expression, when naive bystander T cells were primed in chronically infected hosts. These effects were not T‐cell intrinsic as priming of the naive bystander cells in a naive host restored effector cell differentiation. The transfer of peptide‐loaded DCs restored the proliferative potential of bystander naive cells in chronically infected hosts, which is in line with previous observations where the transfer of peptide‐loaded DCs before tumor inoculation in chronically infected hosts restored granzyme B expression in tumor‐specific CD8 T cells. Together, these data indicate that naive T‐cell impairment in chronically infected hosts is related to T‐cell extrinsic factors.^10^

We observed that naive bystander CD8 T cells express higher levels of CD44, which is a marker that has been associated with memory cells. Also in chronic HCV infection, a larger proportion of naive bystander CD8 T cells has the central memory CD45RA^−^/CD27^+^ phenotype.^9^ CD5 has been correlated with the threshold of T‐cell activation,^21^ where low levels of CD5 promote TCR reactivity. Naive bystander cells in chronic LCMV infection express diminished levels of CD5, suggesting that these cells have increased responsiveness towards cognate antigen, which is also observed in HCV infection.^9^ We did not observe increased proliferation upon priming of the naive bystander cells in chronically infected hosts but rather hampered expansion. However, these effects were due to T‐cell extrinsic factors. Increased proliferation was also not observed when the naive bystander cells isolated from chronically infected hosts were transferred and primed within new hosts. It is possible that CD5 levels are quickly restored when removed from the chronic stimulus, as also anergic cells that express high levels of CD5 quickly exhibit reduced CD5 expression when transferred into an antigen free host.^22^

Combined, this knowledge is very relevant not only in the context of how persistent viruses affect immune homeostasis but also when predicting vaccine responsiveness or immune competence to control heterologous infections in chronically infected hosts.

## CONFLICT OF INTERESTS

The authors declare that there are no conflict of interests.

## AUTHOR CONTRIBUTIONS

IB, SPMW, NJ, RS, and AO designed the experiments. IB, SPMW, MB, NB, KP, and RS performed the experiments. IB, SPMW, and AO analyzed the experiments. IB, SPMW, RS, and AO wrote the manuscript.

## Supporting information

Supporting information

Supporting information

Supporting information

## Data Availability

The data that support the findings of this study are available from the corresponding author upon reasonable request.
