# Supplementary figures and images for: Chronic viral infections impinge on naive bystander CD8 T cells
Source: Immun Inflamm Dis. 2020 Mar 26;8(3):249–57. doi: 10.1002/iid3.300 (PMC7416038; doi:10.1002/iid3.300)

Figure S1

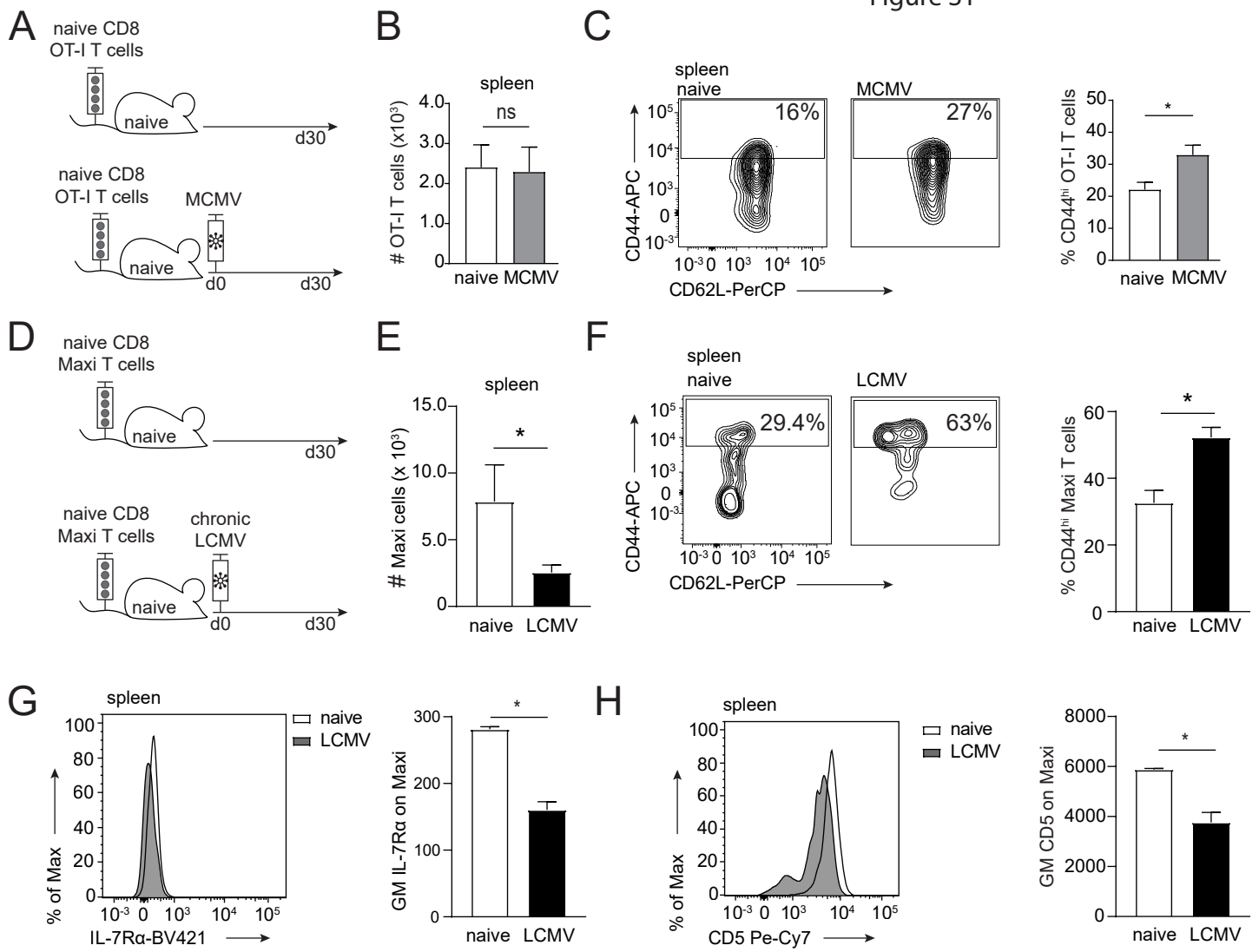

Supplement: Supplementary file 1 — Supporting information [file IID3-8-249-s001.pdf]

Figure S2

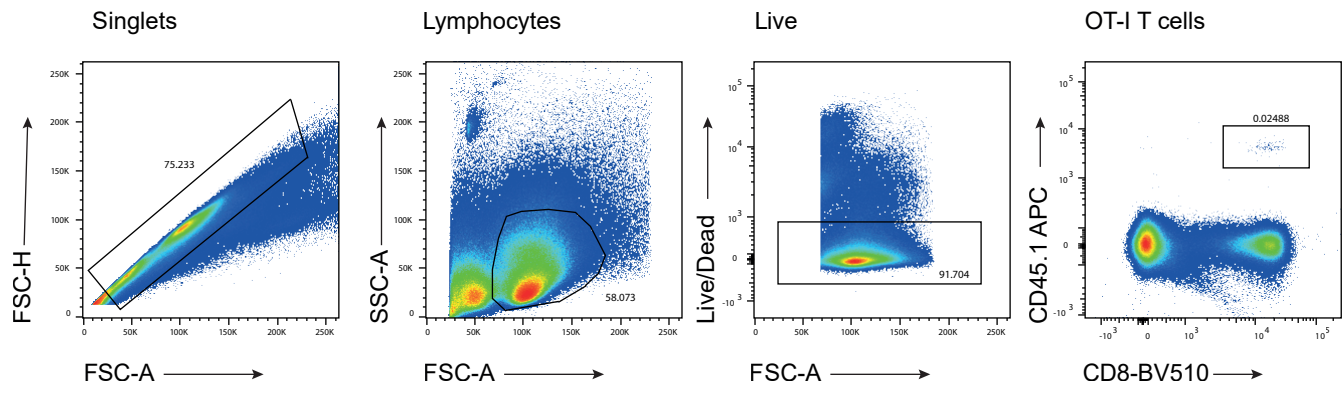

Supplement: Supplementary file 2 — Supporting information [file IID3-8-249-s002.pdf]
