# Supplementary material for: Chronic viral infections impinge on naive bystander CD8 T cells
Source: Immun Inflamm Dis. 2020 Mar 26;8(3):249–57. doi: 10.1002/iid3.300 (PMC7416038; doi:10.1002/iid3.300)
Supplement: Supplementary file 3 — Supporting information [file IID3-8-249-s003.docx]

**Supporting Information**

**Figure S1.** *Chronic virus infections alter naive bystander T cells.*

A) Experimental setup: 10^6^ naive OT-I T cells were adoptively transferred into wild-type mice, followed by an infection with 10^6^ PFU MCMV. B) Number of OT-I T cells in the spleen at day 30 post infection. C) Expression of CD44/CD62L on OT-I T cells. Bar graph represents percentages of OT-I T cells in the spleen that are CD44^hi^. B-C, data from overall 13-14 mice pooled from 3 experiments is shown. D) Experimental setup: 10^6^ naive CD45.1^+^ Maxi CD8 T cells were transferred into hosts that were chronically infected with LCMV the following day. E) Number of Maxi cells in the spleen at day 30 post infection. One experiment out of 3 is shown with 3-5 mice/group/experiment. F) FACS plots show expression of CD44 and CD62L on Maxi cells in the spleen. Bar graph shows percentage of Maxi cells that are CD44^hi^. Pooled data from three experiments is shown with 3-5 mice/group/experiment. G+H) IL7Rα and CD5 expression on Maxi CD8 T cells is shown. Bar graphs show geometric mean of IL7Rα and CD5 on Maxi cells. Data is shown from 1 out of 2 experiment with 3 mice/group/experiment. Bar graphs represent mean + SEM. Statistical analysis was determined using Mann-Whitney test (B-F) or unpaired t-test (G, H), * p<0.05 and ns; p ≥ 0.05.

**Figure S2.** *Flow cytometry gating strategy.*

Representative flow cytometry plots depict the gating strategy to detect OT-I T cells. Doublets were first excluded, followed by selection of lymphocytes and exclusion of death cells using the live/dead marker Near-IR. OT-I T cells were defined by CD45.1^+^/CD8^+^.
